# A Turn-On Detection of DNA Sequences by Means of Fluorescence of DNA-Templated Silver Nanoclusters via Unique Interactions of a Hydrated Ionic Liquid

**DOI:** 10.3390/molecules23112889

**Published:** 2018-11-06

**Authors:** Ye Teng, Hisae Tateishi-Karimata, Takaaki Tsuruoka, Naoki Sugimoto

**Affiliations:** 1 Frontier Institute for Biomolecular Engineering Research (FIBER), Konan University, 7-1-20 Minatojima-Minamimachi, Chuo-ku, Kobe 650-0047, Japan; yteng@center.konan-u.ac.jp (Y.T.); tateishi@konan-u.ac.jp (H.T.-K.); 2 Graduate School of Frontiers of Innovative Research in Science and Technology (FIRST), Konan University, 7-1-20 Minatojima-Minamimachi, Chuo-ku, Kobe 650-0047, Japan; tsuruoka@konan-u.ac.jp

**Keywords:** ionic liquid, choline dihydrogen phosphate, DNA-Ag NCs, DNA detection

## Abstract

Nucleic acid stability and structure, which are crucial to the properties of fluorescent DNA-templated silver nanoclusters (DNA-Ag NCs), significantly change in ionic liquids. In this work, our purpose was to study DNA-Ag NCs in a buffer containing the hydrated ionic liquid of choline dihydrogen phosphate (choline dhp) to improve fluorescence for application in DNA detection. Due to the stabilisation of an i-motif structure by the choline cation, a unique fluorescence emission—that was not seen in an aqueous buffer—was observed in choline dhp and remained stable for more than 30 days. A DNA-Ag NCs probe was designed to have greater fluorescence intensity in choline dhp in the presence of a target DNA. A turn-on sensing platform in choline dhp was built for the detection of the *BRCA1* gene, which is related to familial breast and ovarian cancers. This platform showed better sensitivity and selectivity in distinguishing a target sequence from a mutant sequence in choline dhp than in the aqueous buffer. Our study provides new evidence regarding the effects of structure on properties of fluorescent DNA-Ag NCs and expands the applications of fluorescent DNA-Ag NCs in an ionic liquid because of improved sensitivity and selectivity.

## 1. Introduction

Ionic liquids (ILs), a type of eco-friendly solvents, have garnered interest in recent years because of their unique physicochemical properties such as non-volatility, non-flammability, and good conductivity [1]. ILs have been widely applied, not only in the field of electrochemistry, but also in materials science [2]. For example, nucleic acids are popular building blocks for the design and synthesis of nanomaterials, owing to special base pairing interactions. Unique interactions between nucleic acid and ILs make remarkable differences compared with aqueous conditions [3,4]. A representative IL, choline dihydrogen phosphate (choline dhp), ensures long-term stability of biomolecules, such as proteins and DNA, suggesting that choline dhp is a good solvent for the development of nanomaterials [5,6,7]. Our previous works have revealed different behaviours of nucleic acids in choline dhp [8]. The stability of Watson–Crick and Hoogsteen base pairs is greatly changed in choline dhp [9], in which non-canonical structures such as a triplex and the i-motif are specifically stabilised [10,11]. Thus, the research on nucleic acids in ILs provides new opportunities for the applications of nucleic acid materials in IL environments. Furthermore, ILs have been reported to be good storage media for biomolecules with enhanced stability and activity [12]. Uses of ILs for the synthesis of nanomaterials and for analysis of biological samples are promising, due to the excellent properties of ILs as compared to aqueous systems [13,14,15,16].

As a type of fluorescent DNA nanomaterial, oligonucleotide-templated metal nanoclusters have been a focus of interest for more than 10 years. DNA-encapsulated metal clusters can be obtained via utilisation of the interaction between bases and metal ions such as gold, silver, and copper ions [17]. Specifically, oligonucleotide-templated silver nanoclusters (DNA-Ag NCs) are a class of fluorophores that are especially responsive to optical modulation of their emission. The Ag^+^ ion engages in a strong interaction with a DNA strand, and the addition of sodium borohydride (NaBH_4_) causes several Ag atoms to bind to oligonucleotides, stabilised both locally and globally by the electron-rich nucleobases [18]. Structural and sequence variations of oligonucleotide templates yield specific Ag NCs with distinct emission peaks spanning the blue-green to near-infrared spectral region [19,20,21]. DNA-Ag NCs show great promise as optical reporters for sensitive biological detection, owing to their good solubility, large Stokes shifts, low toxicity, and high emission rates. In particular, specific base pairing and aptamer interactions give DNA materials a natural advantage in the selective detection of various biologically important analytes, and such materials have been employed for the detection of metal ions (Hg^2+^, Cu^2+^) [22,23,24,25], small biomolecules (cysteine, homocysteine, glutathione, and adenosine triphosphate) [26,27], nucleic acids (DNA and RNA) [28,29,30], proteins (thrombin and single-stranded DNA–binding protein) [31,32], and for biological imaging in living cells (intracellular labelling or staining) [33,34,35,36,37]. Nonetheless, the poor stability of DNA-Ag NCs usually limits their applications as compared to Au NCs and quantum dots. The intensity of fluorescent DNA-Ag NCs is gradually quenched after several days, because of oxidation of DNA-Ag NCs [38,39]. The latter phenomenon is thought to be driven by a reaction between the storage solution and the DNA-Ag NCs. This reaction may depend on the structure of the DNA template, although the detailed mechanism is unknown. Moreover, the abundant presence of nucleases decreases the stability of DNA-Ag NCs and greatly limits their practical uses in biological samples. We have shown that choline dhp controls DNA structure and causes nuclease resistance [40]. Thus, choline dhp can be considered an ideal solvent that can alter DNA structures, denature nucleases, and increase the number of applications of DNA-Ag NCs to the turn-on detection of analytes in real samples.

In this work, our purpose was to investigate the improved properties of DNA-Ag NCs in choline dhp. As a result, unique fluorescence emission with excellent stability was observed in DNA-Ag NCs in choline dhp, and a turn-on fluorescence-sensing platform was built for the detection of the *BRCA1* gene, which is related to familial breast and ovarian cancers. 

## 2. Results

### 2.1. C-Ag^+^-C Interaction in Choline Dhp

**Figure 1:**
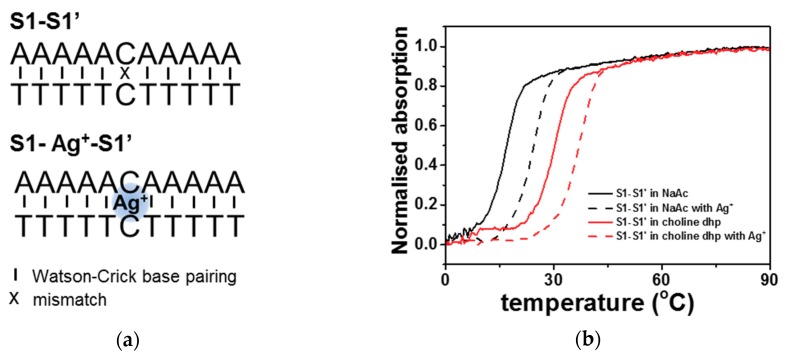
(**a**) The sequences used for the characterisation of stability and proposed base pairing in the absence and presence of Ag^+^ ions. (**b**) The UV melting curves of 2 μM S1-S1′ at 260 nm in the absence or presence of Ag^+^ ions in a buffer consisting of 3.0 M sodium acetate-acetic acid (NaAc-HAc) (pH 7.0) or 3.0 M choline dihydrogen phosphate (dhp)-NaOH (pH 7.0).

Cytosine is reported to bind to the Ag^+^ ion, thus forming a C-Ag^+^-C special interaction [41]. Therefore, C-rich sequences are widely used as templates for the synthesis of DNA-Ag NCs. To increase the practical usefulness of DNA-Ag NCs in choline dhp, the effect of choline dhp on the stability of C-Ag^+^-C was investigated first. According to our previous studies, the stability of nucleic acids greatly changes in choline dhp. For example, A-T base pairs are more stable than C-G base pairs in choline dhp, and i-motif structures are greatly stabilised [9]. The hybrid duplex (S1-S1′) of S1 (5′-AAAAACAAAAA-3′) and S1′ (5′-TTTTTCTTTTT-3′) was designed for characterisation of stability of the C-C mismatch (Figure 1a), which is defined here as one C-C mismatch in the middle and five complementary A-T base pairs on both sides. A high concentration of choline dhp (3.0 M) was chosen to ensure good characteristics of an IL. The stability of duplex structures was characterised by a UV melting assay at 260 nm. Figure 1b shows the melting curves in the absence or presence of Ag^+^ ions in an aqueous solution [3.0 M sodium acetate-acetic acid (NaAc-HAc) buffer (pH 7.0)] and in 3.0 M choline dhp-NaOH (pH 7.0), respectively. The ratio of the concentrations of DNA S1, S1′, and Ag^+^ ions was 1:1:1. In 3.0 M NaAc-HAc (pH 7.0), the melting temperatures of S1-S1′ in the absence and presence of Ag^+^ ions were 17.0 and 24.5 °C, respectively. In 3.0 M choline dhp-NaOH (pH 7.0), the melting temperatures of S1-S1′ in the absence and presence of Ag^+^ ions were 30.4 and 37.0 °C, respectively. According to the *T*_m_ results, S1-S1′ and S1-Ag-S1′ both stabilised in choline dhp, showing higher *T*_m_ values than in the NaAc-HAc buffer. The stabilisation of the duplex is likely related to the binding of choline cations to the minor groove of the duplexes [9]. Then, the effects of environmental conditions on the C-Ag^+^-C interaction could be characterised by means of Δ*T*_m_. These values were calculated from the differences between *T*_m_ values in the absence and presence of Ag^+^ ions (Table S1). In the NaAc-HAc buffer, Δ*T*_m_ was 7.5 °C. In choline dhp-NaOH buffer, Δ*T*_m_ was 6.6 °C, which was 0.9 °C lower than that in the NaAc-HAc buffer. The increases in C-Ag^+^-C stability with Ag^+^ binding were not significantly different in both solutions, indicating that Ag^+^ ions could still be used to stabilise the C-C mismatch in choline dhp. Therefore, C-rich sequences were still assumed to be ideal templates for Ag NCs’ protection in choline dhp. 

### 2.2. DNA-Ag NCs in Choline Dhp

**Figure 2:**
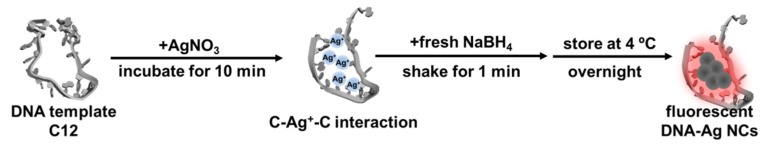
Illustration of the synthesis of C12-templated Ag nanoclusters (NCs).

**Figure 3:**
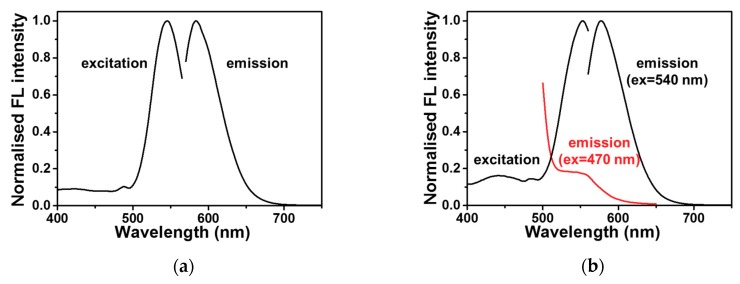
(**a**) The normalised fluorescence (FL) excitation and emission spectra of synthesised C12-templated Ag nanoclusters (NCs) in 3.0 M sodium acetate-acetic acid (NaAc-HAc) (pH 7.0) at 25 °C; (**b**) the normalised FL excitation and emission spectra of C12-Ag NCs in 3.0 M choline dihydrogen phosphate (dhp)-NaOH (pH 7.0) at 25 °C.

Because the Ag^+^ ion stabilised the C-C mismatch in choline dhp, the properties of DNA-Ag NCs were then studied in the buffer containing choline dhp. The fluorescent DNA-Ag NCs were synthesised in an aqueous solution of 0.2 M phosphate buffer (pH 7.0), on the basis of the literature data [18,42], as illustrated in Figure 2. C12 (5′-CCCCCCCCCCCC-3′) was utilised as the template and is the most common template used in the synthesis of DNA-Ag NCs for a DNA detection system. Then, the synthesised C12-Ag NCs were diluted in 3.0 M NaAc-HAc (pH 7.0). After that, 590 nm red fluorescence emission was detected when excitation wavelength was 540 nm (Figure 3a). It is worth nothing that when the synthesised C12-Ag NCs were diluted in 3.0 M choline dhp-NaOH (pH 7.0), the red emission upon excitation at 540 nm shifted from 590 to 577 nm (Figure 3b). In addition, in the excitation spectra, a weak excitation peak was found near 450 nm that was not observed in NaAc-HAc buffer. Because the two emission peaks partially overlapped, and the emission at 577 nm was much stronger, it was difficult to obtain the exact excitation spectra of the emission at 557 nm (Figure S1). The excitation wavelength was determined from the observed maximum fluorescence intensity, which was observed at 557 nm after excitation at 470 nm (Figure 3b, red). The intensity of the emission at 557 nm was only 20% of the strong emission at 577 nm. The fluorescent C12-Ag NCs were next tested as the probe for DNA detection in choline dhp.

Before the use of C12-Ag NCs in choline dhp for DNA detection, we firstly optimised the concentration of choline dhp to attain better properties of fluorescent C12-Ag NCs. Therefore, the fluorescence of C12-Ag NCs in 0, 0.1, 0.3, 0.5, 1.0, 2.0, and 3.0 M choline dhp was measured (Figure S2a). The fluorescence excited at 540 nm shifted to 577 nm, and was greatly enhanced in choline dhp, with maximum intensity in 1.0 M choline dhp (Figure S2b). Higher concentrations of choline dhp quenched the fluorescence emission at 577 nm; this phenomenon might be caused by the high ionic strength, making this IL unsuitable for further applications. Emission at 557 nm after excitation at 470 nm also was noted in choline dhp, and the intensity increased with the increasing concentrations of choline dhp. Therefore, the optimal medium for DNA-Ag NCs was 1.0 M choline dhp, which was next utilised for construction of the DNA-sensing platform in choline dhp. 

**Figure 4:**
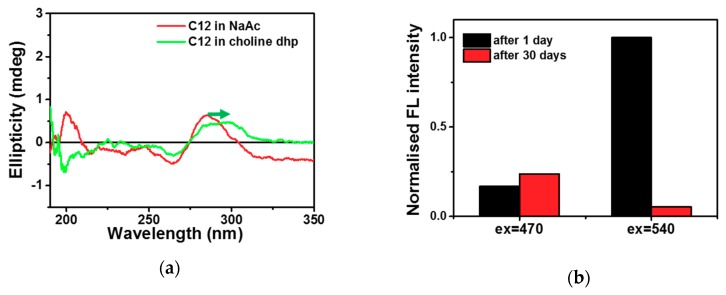
(**a**) The circular dichroism (CD) spectra of 2 μM C12 in 3.0 M sodium acetate-acetic acid (NaAc-HAc) (pH 7.0) and 3.0 M choline dihydrogen phosphate (dhp)-NaOH (pH 7.0) at 25 °C, respectively. (**b**) The normalised emission intensity of C12-templated Ag nanoclusters (NCs) excited at 470 nm (ex = 470) and 540 nm (ex = 540) in 1.0 M choline dhp buffer at 25 °C measured at 1 day and 30 days after the synthesis.

**Figure 5:**
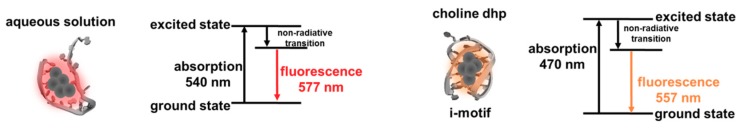
The possible folding of C12-templated Ag nanoclusters (NCs) in an aqueous solution and in choline dihydrogen phosphate (dhp).

The fluorescence of DNA-Ag NCs was sensitive to the templates in the surrounding conditions, depending on the sequences and structures of the template DNAs. As we have shown above in Figure 1b, the *T*_m_ values of S1-S1′ and S1-Ag^+^-S1′ were 17.0 and 24.5 °C, with Δ*T*_m_ of 7.5 °C in the aqueous solution. The *T*_m _values of S1-S1′ and S1-Ag^+^-S1′ were 30.4 and 37.0 °C, with Δ*T*_m_ of 6.6 °C in choline dhp. The similarity of Δ*T*_m_ values indicated that the stability of the C-Ag^+^-C interaction was similar between choline dhp and in aqueous solution, although higher *T*_m_ values of S1-S1′ and S1-Ag^+^-S1′ were obtained due to stabilisation of A-T base pairing in choline dhp. The change of fluorescent properties of C12-Ag NCs was not caused by stabilisation of the C-Ag^+^-C interaction. On the other hand, it has been reported that the i-motif structure is stabilised in choline dhp by the interaction between the choline cation and narrow groove in the i-motif [10], which likely plays an important role in the change of fluorescent properties of C12-Ag NCs. For example, C-rich sequences can fold into i-motif structures with unusual cytosine–cytosine bonds, especially under acidic conditions [43]. In aqueous solutions, the properties of fluorescent DNA-Ag NCs have been reported to be altered by changes in pH [44]. To confirm the possible structural change in choline dhp, the structures of C12 in 3.0 M NaAc buffer and choline dhp were characterised by circular dichroism (CD) spectroscopy as presented in Figure 4a. In NaAc buffer, C12 assumed a single-stranded structure. By contrast, in choline dhp, the positive peak at 280 nm underwent a red shift to 290 nm (the arrow in Figure 4a). The characteristic positive peak at 290 nm indicated the presence of possible i-motif structures, suggesting that C12 in choline dhp was a mixture of single-stranded and i-motif structures. The template DNA structures could then affect the oxidation and aggregation of C12-Ag NCs in solutions; these phenomena are related to the stability of the fluorescent C12-Ag NCs. Moreover, the fluorescence emission at excitation wavelengths 470 and 540 nm in C12-Ag NCs showed different stability in choline dhp, as shown in Figure 4b. The fluorescence emission at 557 nm (upon excitation of Ag NCs at 470 nm), which was present only in the choline dhp group, was relatively weak, but it remained stable for 30 days owing to the stabilisation of the i-motif by choline dhp. On the contrary, the fluorescence emission at 577 nm after excitation at 540 nm got almost fully quenched after 30 days, although the fluorescence emission at 577 nm was strong immediately after the synthesis. The fluorescence emission at 470 nm excitation appeared only in choline dhp; therefore, this fluorescence might have been generated by intramolecular or intermolecular i-motif folding of the template sequence. The changes of fluorescent C12-Ag NCs in choline dhp were assumed to be related to the structure of the DNA template as depicted in Figure 5. Moreover, the intensity of fluorescence upon 470 nm excitation increased by ~40%, whereas the intensity of fluorescence upon excitation at 540 nm decreased by ~90%, suggesting that the fluorescence transition of C12-Ag NCs might be induced by the structural transition from the single-stranded form to the i-motif during storage. Considering that the fluorescence of most DNA-Ag NCs diminished to less than 50% after 48 h of storage in the aqueous solution [45], choline dhp was a good solvent for fluorescence emission at 577 nm after excitation at 540 nm.

### 2.3. Detection of DNA by Means of DNA-Ag NCs in Choline Dhp

**Figure 6:**
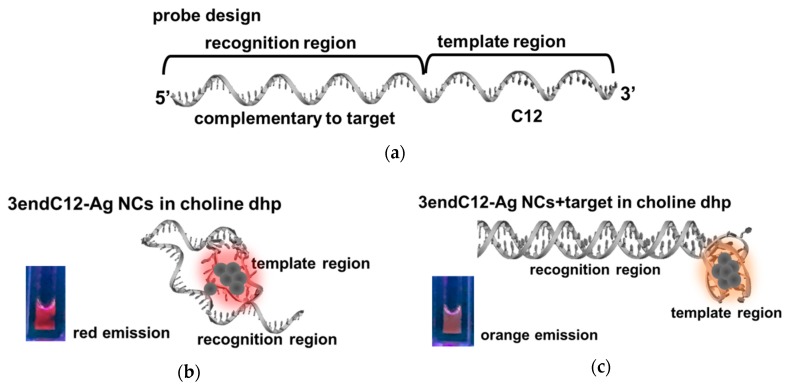
(**a**) The design of the probe and a photo of 3endC12-templated Ag nanoclusters (NCs) (**b**) in the absence and (**c**) presence of a target (the photo was taken during UV light excitation at 254 nm).

The DNA-Ag NCs were next applied to the detection of a DNA sequence in choline dhp. A part of the DNA sequence of the *BRCA1* breast cancer gene (Table 1, target) was chosen as the target in this work, because it has been reported that the replacement of guanine (G) with thymine (T) in this gene (Table 1, mutant) is linked to familial breast and ovarian cancers [46,47]. 3endC12 (Table 1) was the designed template for Ag NC synthesis, and contains a complementary part of the target sequence for recognition as well as a C12 template at the 3′ end for the encapsulation of Ag NCs (Figure 6a). 

**Figure 7:**
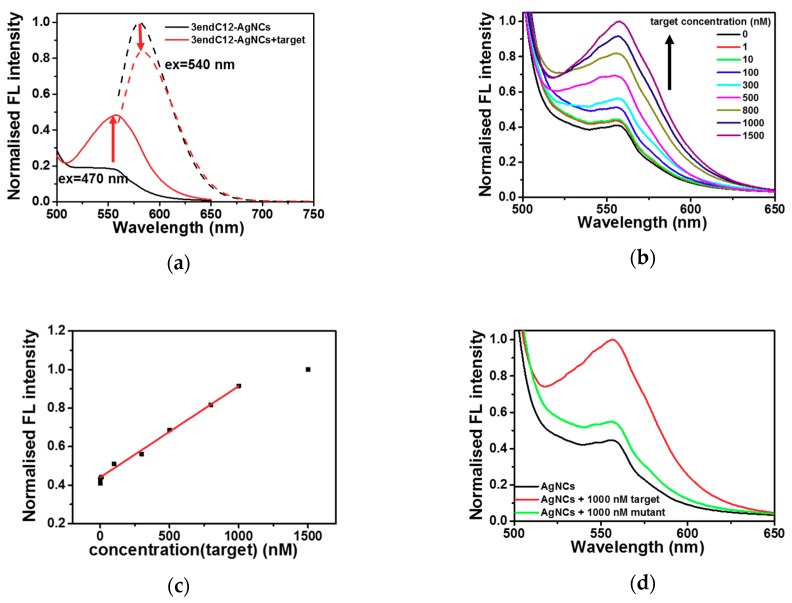
(**a**) The fluorescence spectra of 1 μM 3endC12-templated Ag nanoclusters (NCs) excited at 470 nm (solid curve) and 540 nm (dashed curve) at 25 °C in the absence (black) and presence (red) of 1 μM target in 1.0 M choline dihydrogen phosphate (dhp) (pH 7.0). (**b**) The fluorescence spectra of 3endC12-Ag NCs excited at 470 nm at 25 °C at different concentrations of the target. (**c**) The relation between fluorescence emission intensity at 557 nm after 470 nm excitation at 25 °C with the concentration of the target. (**d**) The fluorescence spectra of 3endC12-Ag NCs excited at 470 nm and 25 °C in the absence or presence of 1000 nM target or mutant.

According to the results above, the structural change in DNAs can induce specific fluorescent properties. Figure 6b,c show the 3endC12-Ag NCs in choline dhp under UV light. In the absence of a target, strong red emission was observed when the solution was excited at 254 nm by the UV lamp (Figure 6b). An obvious colour change was detected after addition of a hybrid target sequence (Figure 6c), which could be distinguished under UV light. The phenomenon was next confirmed by analysis of fluorescence spectra. As depicted in Figure 7a, in the presence of the target, the fluorescence emission at 540 nm excitation decreased, while the fluorescence emission at 557 nm after 470 nm excitation increased. These changes resulted in a colour change from red to orange under UV light excitation at 254 nm, and were then utilised for construction of the sensing platform for the DNA target. Nevertheless, the strong fluorescence emission at 577 nm after 470 nm excitation showed poor correlation with target concentrations after further study (Figure S3), indicating that it is not suitable for DNA detection. However, the fluorescence emission at 557 nm after 470 nm excitation in the choline dhp condition manifested good stability and greater intensity relative to the concentration of the target. A comparison of fluorescent properties of different species of Ag NCs is given in Table 2, and the fluorescence emission at 557 nm after 470 nm excitation in the choline dhp solution was then applied to the detection of the DNA target.

Under the optimal conditions, different concentrations of the target DNA in the range 1 to 1500 nM were added to the 3endC12-Ag NCs. The fluorescence spectra are presented in Figure 7b. The fluorescence of 3endC12-Ag NCs at 557 nm after 470 nm excitation increased in the presence of the target and manifested a good linear relation in a range of 1 to 1000 nM (R^2^ = 0.997, Figure 7c). The lowest concentration of target DNA that could be detected was 1 nM. The ΔI calculated from the normalized fluorescence intensity in the presence and absence of 1 nM target was 0.0255, which was 6.5 times the standard deviation of 0.0039 calculated from the measured fluorescence intensity of blank samples. Therefore, the limit of detection (LOD) of our method was 1 nM, which can be significantly distinguished from the background fluorescence of probe Ag NCs. The sensing system in choline dhp achieved detection of DNA via fluorescence emission at 557 nm after excitation at 470 nm. This type of fluorescence did not exist in the aqueous buffer and yielded an LOD that was better (lower) than the LOD of 14 nM in the aqueous solution [17]. In addition, as illustrated in Figure 7d, the presence of the mutant sequence, which contained one mismatch (Table 1), induced only 10% fluorescence enhancement when compared to the full-match target at the same concentration, indicating that the sensing method had significant selectivity to distinguish between a fully matching target and the mismatched mutant sequence. The sensitivity (LOD) and selectivity of our method are comparable to those of the existing detection methods [28,30].

## 3. Discussion

DNA-Ag NCs as novel fluorescent nanomaterials have been utilised for the detection of many biomolecules. However, there are still many open questions. The properties of fluorescent DNA-Ag NCs have been reported to strongly depend on the template sequences, and the excitation and emission wavelengths can be altered simply by changing the sequences of templates [8]. Moreover, DNA-Ag NCs are sensitive to changes in their surrounding environments, for example, the use of G-rich sequences and other C-rich templates via hybridisation [9,27]. Nonetheless, the mechanism of environment-induced fluorescence change is not well understood. Currently, the exact relation between the properties of fluorescent DNA-Ag NCs and the characteristics of templates (including sequences and structures) is unknown. At present, we cannot design a template sequence with specific fluorescent emission wavelengths as needed. More information about how DNA structures affect the properties of fluorescent DNA-Ag NCs can help researchers understand the mechanisms of DNA-Ag NCs’ formation and the regulation of fluorescent properties. 

ILs are promising solvents for regulation of the structures of nucleic acids and might further optimise the properties of fluorescent DNA-Ag NCs. Choline dhp in particular has been reported to significantly alter the stability of nucleic acids via binding in the grooves of DNAs [8]. For example, the triplex and i-motif structures are important for the interactions between a DNA template and Ag atoms in Ag NCs. Moreover, stabilisation of the duplex is crucial for the recognition of target sequences and is likely to improve selectivity of sensing methods in choline dhp. In other studies, the emission wavelengths of DNA-Ag NCs when tested with i-motif–forming templates have been reported to be shifted when the structures of templates are regulated by pH and the loop sequence [44,48]. In the present work, the CD spectra revealed that in choline dhp, the i-motif structure stabilises, in contrast to the aqueous solution. The i-motif structures when excited at 470 nm yielded fluorescence emission at 557 nm with good stability for 30 days. These results can greatly expand the practical uses of DNA-Ag NCs. The stable i-motif–templated Ag NCs can be employed to construct a sensitive turn-on detecting platform with good selectivity. Stability, sensitivity, and selectivity all showed better values in choline dhp, suggesting that DNA-Ag NCs may be a group of promising fluorescent probes for nucleic acids, small biomolecules, and proteins in ILs, especially for the detection of analytes in real samples. In this study, as illustrated in Figure 6b, Ag atoms were more likely to cluster in the C12 template region during the synthesis of 3endC12-Ag NCs, while a small amount of Ag atoms may bind to cytosine or thymine in the recognition region [49]. The formation of a compact i-motif structure within the template region in choline dhp allowed the recognition region to bind more freely to the target strand (Figure 6c). Hybridisation of the recognition region with complementary target DNA was supposed to facilitate the clustering in template region and induced the fluorescence signal change. 

Furthermore, the environmental conditions caused a significant structural transition, further affecting the properties of fluorescent Ag NCs as shown in Figure 4. In the aqueous buffer, Ag^+^ preferred to bind to cytosine via the C-Ag^+^-C interaction. After the reduction reaction, fluorescent Ag NCs were obtained, due to size-quantised discrete energy levels. Nevertheless, this situation resulted in a relatively weak structure that was hardly stable for a long time. The flexible single-stranded template might have influenced the stability of Ag NCs, which dispersed on the template without apparent symmetry. In choline dhp, the i-motif was greatly stabilised by choline cations. The template part formed a highly ordered secondary structure of the i-motif, which was more symmetrical and stable in choline dhp. The specific structure of the i-motif may lead a symmetric distribution of Ag atoms across the template, thereby stabilising the fluorescence of Ag NCs. In addition, due to the i-motif folding, the recognition motif was free to combine with the target, thus improving sensitivity and selectivity of the method in choline dhp. Moreover, in choline dhp, the dielectric constant of the surrounding environment is changed; this alteration also influenced the properties and stability of fluorescent DNA-Ag NCs. Thus, choline dhp provides an ideal environment to regulate DNA template structures and to study the relation between DNA structures and properties of fluorescent DNA-Ag NCs in detail. Strategies taking advantage of base pairing and structure transition for the design of DNA sensors can be applied under choline dhp conditions, although we should pay attention to the potential interference of an additional fragment in the design of a probe template. The improvement of the properties of fluorescent DNA-Ag NCs in choline dhp may further increase the number of practical applications for the detection of analytes in real samples. 

## 4. Materials and Methods 

### 4.1. Chemicals and Materials

All the oligodeoxynucleotides employed in this work were purchased from Japan Bio Services Co. (Saitama, Japan) and were used without further purification. The sequences are listed in Table S1. All the DNA samples were dissolved in double-distilled water purified by a Milli-Q system (Millipore Corporation, Burlington, MA, USA). Concentrations of DNAs were determined via absorbance at 260 nm and 90 °C. NaBH_4_, silver nitrate (AgNO_3_), and other salts were acquired from Sigma-Aldrich (St. Louis, MO, USA). Choline dhp was bought from Ionic Liquids Technologies Co., Ltd., (Heilbronn, Germany) and used without further purification. 

### 4.2. The UV Melting Assay

UV-Vis absorption spectra were recorded on a Shimadzu 1700 spectrophotometer (Kyoto, Japan) equipped with a thermoprogrammer. The stability of DNA was evaluated by changes in UV melting temperatures. In particular, thermal melting of a 10 μM DNA sample was analysed in a buffer consisting of 3.0 M NaAc-HAc or choline dhp at pH 7.0 in the absence or presence of 10 μM Ag ions. Samples were incubated at 90 °C for 3.0 min followed by cooling from 90 to 0 °C at a rate of −0.5 °C·min^−1^. The samples were melted from 0 to 90 °C at a rate of 0.5 °C·min^−1^.

### 4.3. Synthesis of DNA-Ag NCs

Single-stranded DNA 3endC12 (5′-GATTTTCTTCCTTTTGTTCCCCCCCCCCCCC-3′) is composed of a C-rich template (CCCCCCCCCCCC) for Ag cluster formation at the 3′ end and a specific DNA sequence (GATTTTCTTCCTTTTGTTC) for recognition of a target DNA. DNA-Ag NCs were prepared according to other reports [18,42]. Briefly, 60 μM AgNO_3_ was added to 10 μM DNA (dissolved in sodium phosphate buffer; 200 mM, pH 7.0). After incubation at room temperature for 10 min, fresh 60 μM NaBH_4_ was added to the mixture to reduce Ag^+^ at the molar ratio DNA:Ag^+^:NaBH_4_ of 1:6:6, and the resultant solution was vigorously shaken for 1 min. After that, the reaction mixture was incubated in the dark at 4 °C overnight. The synthesized DNA-Ag NCs were diluted with indicated buffers without further purification in the experiments for detection of DNA. 

### 4.4. Detection Methods in Choline Dhp

In this work, detection experiments were carried out at 25 °C, unless specified otherwise. The synthesised 3endC12-templated Ag NCs were diluted with 1.0 M choline dhp-NaOH (pH 7.0) to a concentration of 1 μM. The diluted 3endC12-templated Ag NCs were employed for the detection of the DNA target sequence. The target sequence can completely hybridise with the recognition portion of 3endC12. Different concentrations of targets were added to the 1 μM solution of DNA-Ag NCs, followed by incubation at 40 °C before the fluorescence measurement.

## 5. Conclusions

Extra fluorescence emission from DNA-Ag NCs was observed in choline dhp owing to the structural change of DNA templates. Possibly, i-motif structures formed within the template in choline dhp. The fluorescent DNA-Ag NCs in choline dhp were applied to the design of a DNA-sensing platform. A good linear relation was obtained in a range of 1 to 1000 nM, with good selectivity. A mutant target sequence produced much weaker signals. The sensing method of DNA-Ag NCs in choline dhp is simple, sensitive, and selective, and the resulting fluorescence shows better stability in choline dhp than in an aqueous solution. Promising practical applications of DNA-Ag NCs in green and sustainable ILs are expected in the future.

## Figures and Tables

**Table 1:** Sequences used for DNA detection in this work.

Name	Sequences (5′-3′)
target	GAACAAAAGGAAGAAAATC
mutant ^1^	GAACAAAAGGAA**T**AAAATC
3endC12 ^2^	GATTTTCTTCCTTTTGTTCCCCCCCCCCCCC

^1^ The mismatched base in mutant sequence is boldfaced. ^2^ The recognition region for the target sequence is underlined.

**Table 2:** A comparison of fluorescent properties of 3endC12-Ag NCs between the aqueous solution and choline dhp.

Excitation (nm)/Emission (nm)	In Aqueous Solution			In Choline Dhp		
	Fluorescence	Stability	Sensing	Fluorescence	Stability	Sensing
470/557	-	-	-	weak	good	suitable
540/577	strong	poor	unsuitable	strong	poor	unsuitable

## Supplementary Materials

The following are available online. Figure S1: The excitation spectrum of C12-Ag NCs at 557 nm in 3.0 M choline dhp (pH 7.0) at 25 °C. Figure S2. (a) The fluorescence spectra of C12-Ag NCs excited at 470 nm (solid) and 540 nm (dash) in the buffers containing 0, 0.1, 0.3, 0.5, 1.0, 2.0 and 3.0 M choline-dhp-NaOH (pH 7.0) at 25 °C. (b) The normalized fluorescence intensity of C12-Ag NCs of 557 nm at 470 nm excitation (black) and of 577 nm at 540 nm excitation (red) in the buffers containing 0, 0.1, 0.3, 0.5, 1.0, 2.0 and 3.0 M choline-dhp-NaOH (pH 7.0) at 25 °C. Figure S3. (a) The fluorescence spectra of 3endC12-Ag NCs at 25 °C excited at 540 nm with different concentrations of target. (b) The relationship between fluorescence intensity of 577 nm at 540 nm excitation with the concentration of target. Table S1: The melting temperature of S1-S1′ in the absence and presence of silver ions in a buffer containing 3.0 M NaAc-HAc (pH 7.0) and 3.0 M choline dhp-NaOH (pH 7.0) at 25 °C.

## References

[1] Earle M.J., Seddon K.R. (2000). Ionic liquids. Green solvents for the future. Pure Appl. Chem..

[2] Tateishi-Karimata H., Sugimoto N. (2018). Biological and nanotechnological applications using interactions between ionic liquids and nucleic acids. Biophys. Rev..

[3] Chandran A., Ghoshdastidar D., Senapati S. (2012). Groove Binding Mechanism of Ionic Liquids: A Key Factor in Long-Term Stability of DNA in Hydrated Ionic Liquids?. J. Am. Chem. Soc..

[4] Saha D., Mukherjee A. (2018). Effect of water and ionic liquids on biomolecules. Biophys. Rev..

[5] Vijayaraghavan R., Izgorodin A., Ganesh V., Surianarayanan M., MacFarlane D.R. (2010). Long-term structural and chemical stability of DNA in hydrated ionic liquids. Angew. Chem. Int. Ed. Engl..

[6] Mukesh C., Mondal D., Sharma M., Prasad K. (2013). Rapid dissolution of DNA in a novel bio-based ionic liquid with long-term structural and chemical stability: Successful recycling of the ionic liquid for reuse in the process. Chem. Commun..

[7] Fujita K., MacFarlane D.R., Forsyth M., Yoshizawa-Fujita M., Murata K., Nakamura N., Ohno H. (2007). Solubility and Stability of Cytochrome c in Hydrated Ionic Liquids:  Effect of Oxo Acid Residues and Kosmotropicity. Biomacromolecules.

[8] Nakano M., Tateishi-Karimata H., Tanaka S., Sugimoto N. (2014). Choline Ion Interactions with DNA Atoms Explain Unique Stabilization of A-T Base Pairs in DNA Duplexes: A Microscopic View. J. Phys. Chem. B.

[9] Tateishi-Karimata H., Sugimoto N. (2012). A-T Base Pairs are More Stable Than G-C Base Pairs in a Hydrated Ionic Liquid. Angew. Chem. Int. Ed..

[10] Tateishi-Karimata H., Nakano M., Pramanik S., Tanaka S., Sugimoto N. (2015). i-Motifs are more stable than G-quadruplexes in a hydrated ionic liquid. Chem. Commun..

[11] Tateishi-Karimata H., Nakano M., Sugimoto N. (2014). Comparable Stability of Hoogsteen and Watson-Crick Base Pairs in Ionic Liquid Choline Dihydrogen Phosphate. Sci. Rep..

[12] Mazid R.R., Cooper A., Zhang Y., Vijayaraghavan R., MacFarlane D.R., Cortez-Jugo C., Cheng W.L. (2015). Enhanced enzymatic degradation resistance of plasmid DNA in ionic liquids. RSC Adv..

[13] Behera K., Pandey S., Kadyan A., Pandey S. (2015). Ionic Liquid-Based Optical and Electrochemical Carbon Dioxide Sensors. Sensors.

[14] Clark K.D., Trujillo-Rodriguez M.J., Anderson J.L. (2018). Advances in the analysis of biological samples using ionic liquids. Anal. Bioanal. Chem..

[15] Zhang Y.M., Cao Y.H., Chen D., Cui P.L., Yang J. (2018). Ionic liquid assisted synthesis of palladium nanoclusters for highly efficient formaldehyde oxidation. Electrochim. Acta.

[16] Jagannath B., Muthukumar S., Prasad S. (2018). Electrical double layer modulation of hybrid room temperature ionic liquid/aqueous buffer interface for enhanced sweat based biosensing. Anal. Chim. Acta.

[17] Pandya A., Lad A.N., Singh S.P., Shanker R. (2016). DNA assembled metal nanoclusters: Synthesis to novel applications. RSC Adv..

[18] Petty J.T., Zheng J., Hud N.V., Dickson R.M. (2004). DNA-templated Ag nanocluster formation. J. Am. Chem. Soc..

[19] Richards C.I., Choi S., Hsiang J.C., Antoku Y., Vosch T., Bongiorno A., Tzeng Y.L., Dickson R.M. (2008). Oligonucleotide-stabilized Ag nanocluster fluorophores. J. Am. Chem. Soc..

[20] Martinez J.S., Sharma J., Yeh H.C., Yoo H., Werner J.H. (2010). A complementary palette of fluorescent silver nanoclusters. Chem. Commun..

[21] New S.Y., Lee S.T., Su X.D. (2016). DNA-templated silver nanoclusters: Structural correlation and fluorescence modulation. Nanoscale.

[22] Guo W.W., Yuan J.P., Wang E.K. (2009). Oligonucleotide-stabilized Ag nanoclusters as novel fluorescence probes for the highly selective and sensitive detection of the Hg^2+^ ion. Chem. Commun..

[23] Deng L., Zhou Z., Li J., Li T., Dong S. (2011). Fluorescent silver nanoclusters in hybridized DNA duplexes for the turn-on detection of Hg^2+^ ions. Chem. Commun..

[24] Lan G.Y., Huang C.C., Chang H.T. (2010). Silver nanoclusters as fluorescent probes for selective and sensitive detection of copper ions. Chem. Commun..

[25] Chang H.T., Su Y.T., Lan G.Y., Chen W.Y. (2010). Detection of Copper Ions Through Recovery of the Fluorescence of DNA-Templated Copper/Silver Nanoclusters in the Presence of Mercaptopropionic Acid. Anal. Chem..

[26] Wang E.K., Han B.Y. (2011). Oligonucleotide-stabilized fluorescent silver nanoclusters for sensitive detection of biothiols in biological fluids. Biosens. Bioelectron..

[27] Zhou Z., Du Y., Dong S. (2011). DNA-Ag nanoclusters as fluorescence probe for turn-on aptamer sensor of small molecules. Biosens. Bioelectron..

[28] Lan G.Y., Chen W.Y., Chang H.T. (2011). One-pot synthesis of fluorescent oligonucleotide Ag nanoclusters for specific and sensitive detection of DNA. Biosens. Bioelectron..

[29] Petty J.T., Sengupta B., Story S.P., Degtyareva N.N. (2011). DNA Sensing by Amplifying the Number of Near-Infrared Emitting, Oligonucleotide-Encapsulated Silver Clusters. Anal. Chem..

[30] Yang S.W., Vosch T. (2011). Rapid Detection of MicroRNA by a Silver Nanocluster DNA Probe. Anal. Chem..

[31] Martinez J.S., Sharma J., Yeh H.C., Yoo H., Werner J.H. (2011). Silver nanocluster aptamers: In situ generation of intrinsically fluorescent recognition ligands for protein detection. Chem. Commun..

[32] Lan G.Y., Chen W.Y., Chang H.T. (2011). Characterization and application to the detection of single-stranded DNA binding protein of fluorescent DNA-templated copper/silver nanoclusters. Analyst.

[33] Choi S.M., Yu J.H., Patel S.A., Tzeng Y.L., Dickson R.M. (2011). Tailoring silver nanodots for intracellular staining. Photoch. Photobiol. Sci..

[34] Li J., Zhong X., Cheng F., Zhang J.-R., Jiang L.-P., Zhu J.-J. (2012). One-Pot Synthesis of Aptamer-Functionalized Silver Nanoclusters for Cell-Type-Specific Imaging. Anal. Chem..

[35] Yin J., He X., Wang K., Qing Z., Wu X., Shi H., Yang X. (2012). One-step engineering of silver nanoclusters-aptamer assemblies as luminescent labels to target tumor cells. Nanoscale.

[36] Ai J., Guo W., Li B., Li T., Li D., Wang E. (2012). DNA G-Quadruplex-Templated Formation of the Fluorescent Silver Nanocluster and Its Application to Bioimaging. Talanta.

[37] Sun Z., Wang Y., Wei Y., Liu R., Zhu H., Cui Y., Zhao Y., Gao X. (2011). Ag cluster-aptamer hybrid: Specifically marking the nucleus of live cells. Chem. Commun..

[38] Morishita K., MacLean J.L., Liu B., Jiang H., Liu J. (2013). Correlation of photobleaching, oxidation and metal induced fluorescence quenching of DNA-templated silver nanoclusters. Nanoscale.

[39] Li J., Yu J., Huang Y., Zhao H., Tian L. (2018). Highly Stable and Multiemissive Silver Nanoclusters Synthesized in Situ in a DNA Hydrogel and Their Application for Hydroxyl Radical Sensing. ACS Appl. Mater. Interfaces.

[40] Tateishi-Karimata H., Pramanik S., Sugimoto N. (2015). DNA sensor's selectivity enhancement and protection from contaminating nucleases due to a hydrated ionic liquid. Analyst.

[41] Ono A., Cao S., Togashi H., Tashiro M., Fujimoto T., Machinami T., Oda S., Miyake Y., Okamoto I., Tanaka Y. (2008). Specific interactions between silver(i) ions and cytosine-cytosine pairs in DNA duplexes. Chem. Commun..

[42] Teng Y., Yang X., Han L., Wang E. (2014). The Relationship between DNA Sequences and Oligonucleotide-Templated Silver Nanoclusters and Their Fluorescence Properties. Chem.-Eur. J..

[43] Day H.A., Pavlou P., Waller Z.A.E. (2014). i-Motif DNA: Structure, stability and targeting with ligands. Bioorg. Med. Chem..

[44] Sengupta B., Springer K., Buckman J.G., Story S.P., Abe O.H., Hasan Z.W., Prudowsky Z.D., Rudisill S.E., Degtyareva N.N., Petty J.T. (2009). DNA Templates for Fluorescent Silver Clusters and I-Motif Folding. J. Phys. Chem. C.

[45] Sharma J., Rocha R.C., Phipps M.L., Yeh H.-C., Balatsky K.A., Vu D.M., Shreve A.P., Werner J.H., Martinez J.S. (2012). A DNA-templated fluorescent silver nanocluster with enhanced stability. Nanoscale.

[46] Wang J., Kawde A.N. (2001). Pencil-based renewable biosensor for label-free electrochemical detection of DNA hybridization. Anal. Chim. Acta.

[47] Tong D., Stimpfl M., Reinthaller A., Vavra N., Mullauer-Ertl S., Leodolter S., Zeillinger R. (1999). BRCAI gene mutations in sporadic ovarian carcinomas: Detection by PCR and reverse allele-specific oligonucleotide hybridization. Clin. Chem..

[48] Li T.T., He N.Y., Wang J.H., Li S., Deng Y., Wang Z.L. (2016). Effects of the i-motif DNA loop on the fluorescence of silver nanoclusters. RSC Adv..

[49] Nordén B., Matsuoka Y., Kurucsev T. (1986). Nucleic acid-metal interactions. IV. Complexes of Ag(I) with thymine and cytosine from studies of UV and IR dichroic spectra. J. Crystallogr. Spectrosc. Res..

