# Supplementary material for: A Turn-On Detection of DNA Sequences by Means of Fluorescence of DNA-Templated Silver Nanoclusters via Unique Interactions of a Hydrated Ionic Liquid
Source: Molecules. 2018 Nov 6;23(11):2889. doi: 10.3390/molecules23112889 (PMC6278258; doi:10.3390/molecules23112889)
Supplement: Supplementary file 1 [file molecules-23-02889-s001.pdf]

Supporting Information

A turn-on detection of DNA sequences by means of fluorescence of DNA-templated silver nanoclusters via unique interactions of a hydrated ionic liquid

Ye Teng, Hisae Tateishi-Karimata, Takaaki Tsuruoka and Naoki Sugimoto*

Frontier Institute for Biomolecular Engineering Research (FIBER), and Graduate School of Frontiers of Innovative Research in Science and Technology (FIRST), Konan University,

7-1-20 Minatojima-Minamimachi, Chuo-ku, Kobe 650-0047, Japan.

E-mail address: sugimoto@konan-u.ac.jp

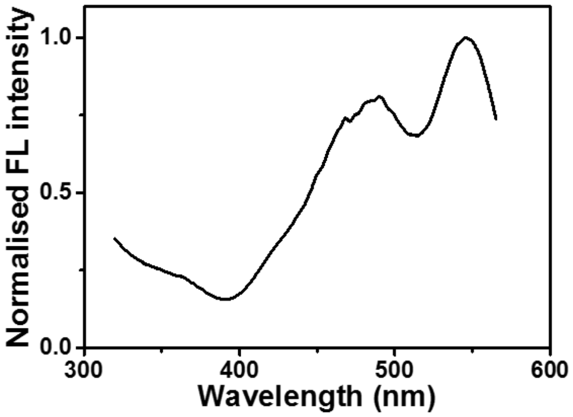

Figure S1. The excitation spectrum of C12-Ag NCs at 557 nm in 3.0 M choline dhp (pH 7.0) at 25 °C.

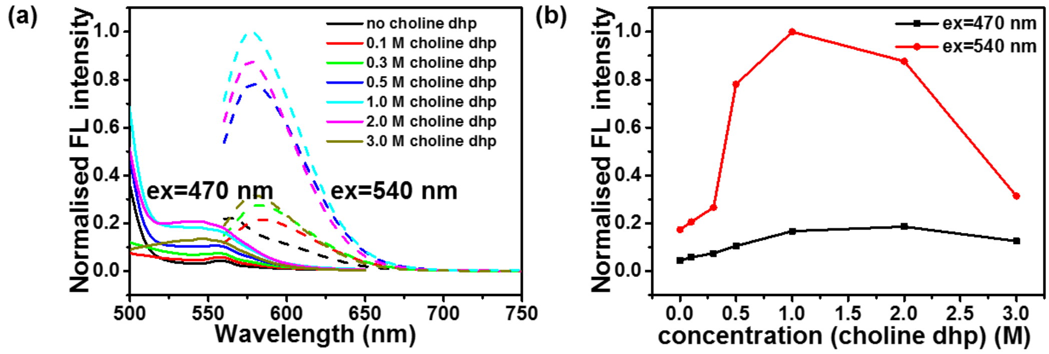

Figure S2. (**a**) The fluorescence spectra of C12-Ag NCs excited at 470 nm (solid) and 540 nm (dash) in the buffers containing 0, 0.1, 0.3, 0.5, 1.0, 2.0 and 3.0 M choline-dhp-NaOH (pH 7.0) at 25 °C. (**b**) The normalized fluorescence intensity of C12-Ag NCs of 557 nm at 470 nm excitation (black) and of 577 nm at 540 nm excitation (red) in the buffers containing 0, 0.1, 0.3, 0.5, 1.0, 2.0 and 3.0 M choline-dhp-NaOH (pH 7.0) at 25 °C.

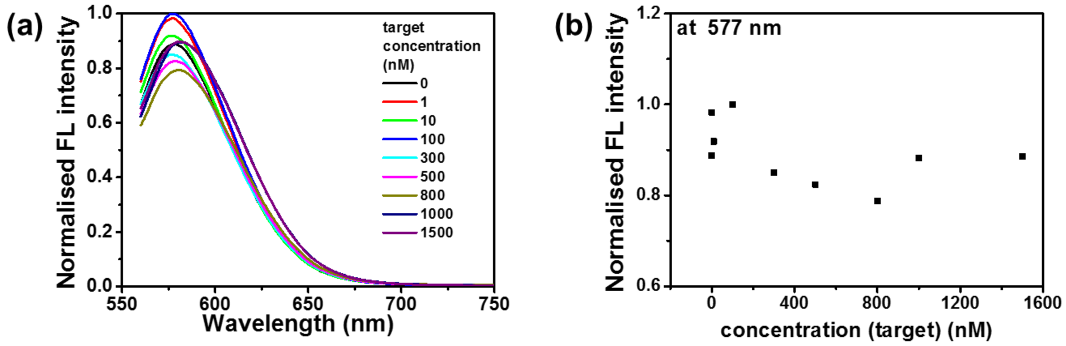

Figure S3. (a) The fluorescence spectra of 3endC12-Ag NCs at 25 °C excited at 540 nm with different concentrations of target. (b) The relationship between fluorescence intensity of 577 nm at 540 nm excitation with the concentration of target.

Table S1. The melting temperature of S1-S1' in the absence and presence of silver ions in a buffer containing 3.0 M NaAc-HAc (pH 7.0) and 3.0 M choline dhp-NaOH (pH 7.0) at 25 °C

	T_m (°C) (NaAc-HAc)	T_m (°C) (choline dhp-NaOH)
S1-S1'	17.0	30.4
S1-S1' + Ag^+	24.5	37.0
ΔT_m (°C)	+7.5	+6.6
